# A new data science trajectory for analysing multiple studies: a case study in physical activity research

**DOI:** 10.1016/j.mex.2024.103104

**Published:** 2024-12-11

**Authors:** Simone Catharina Maria Wilhelmina Tummers, Arjen Hommersom, Catherine Bolman, Lilian Lechner, Roger Bemelmans

**Affiliations:** a Open University of the Netherlands, Heerlen, the Netherlands; b Radboud University, Nijmegen, the Netherlands; c Zuyd University of Applied Sciences, Heerlen, the Netherlands

**Keywords:** Multiple studies, Applied data science, Data Science Trajectories, DST trajectory for data science analysis of multiple studies

## Abstract

The analysis of complex mechanisms within population data, and within sub-populations, can be empowered by combining datasets, for example to gain more understanding of change processes of health-related behaviours. Because of the complexity of this kind of research, it is valuable to provide more specific guidelines for such analyses than given in standard data science methodologies. Thereto, we propose a generic procedure for applied data science research in which the data from multiple studies are included. Furthermore, we describe its steps and associated considerations in detail to guide other researchers. Moreover, we illustrate the application of the described steps in our proposed procedure (presented in the graphical abstract) by means of a case study, i.e., a physical activity (PA) intervention study, in which we provided new insights into PA change processes by analyzing an integrated dataset using Bayesian networks. The strengths of our proposed methodology are subsequently illustrated, by comparing this data science trajectories protocol to the classic CRISP-DM procedure. Finally, some possibilities to extend the methodology are discussed.

– A detailed process description for multidisciplinary data science research on multiple studies.
– Examples from a case study illustrate methodological key points.

## Graphical abstract

**Figure:**
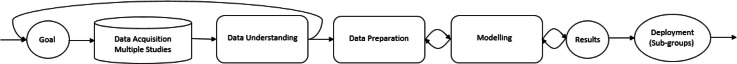

Specifications table

**Table:** 

Subject area:	Computer Science
More specific subject area:	Data science methodologies
Name of your method:	DST trajectory for data science analysis of multiple studies
Name and reference of original method:	1). The CRISP-DM model; Chapman P, Clinton J, Kerber R, Khabaza T, Reinartz T, Shearer C, et al. CRISP-DM 1.0: Step-by-step data mining guide. SPSS inc. 2000;9(13):1-73(Published) [2]. 2). The Data Science Trajectories framework; Martínez-Plumed F, Contreras-Ochando L, Ferri C, Hernández-Orallo J, Kull M, Lachiche N, et al. CRISP-DM twenty years later: From data mining processes to data science trajectories. IEEE Transactions on Knowledge and Data Engineering. 2019;33(8):3048-61(Published) [3].
Resource availability:	Not applicable. No new data were created or analysed in this study.

## Background

Significant data fusion research has been conducted in various domains [4], including in Physical Activity (PA) analysis [1,5]. Single study data often provide limited insight into the structure of variable relations, due to sub-population focus and restricted generalizability. Integrating data from multiple studies improves validity, reliability, understanding of broader phenomena, and empowerment of sub-population research. However, little attention has been given to data mining methodologies for multiple datasets, and general methodology descriptions rarely address the implementation of steps.

In general, multidisciplinary research that integrates similar variables measured in comparable studies and applies non-traditional data science techniques, can reveal a more robust insight into complex problems. In addition, combining datasets can empower the analyses. In such a way, new insights can be gained about relevant (sub-population specific) mechanisms. For example, in recent research (the case study), integrating data from several studies enhanced statistical power and generalizability [1]. This ultimately led to new findings on PA behavior change, including gender-specific insights. Training machine learning models on this combined dataset provided valuable results, particularly in assessing the impact of online interventions on actual PA behaviour change. Associated single studies examining pathways of effect relations and predictors of change in PA e-health intervention provided limited insights [[6], [7], [8]]. Note that in these studies traditional statistical analysis techniques have been applied, such as (multilevel) logistic and linear regression, including mediation or moderation analyses [[9], [10], [11], [12]]. Because of its added value and complexity, it would be relevant to get insight into what such multidisciplinary research analysing data from multiple studies on the same phenomenon looks like, in order to facilitate similar future research. Therefore, a systematic outline of the data mining process is desirable, along with detailed descriptions of associated obstacles and considerations to guide researchers. A case study involving data science and (health) psychology can illustrate the execution of the methodology steps effectively.

There are various methodologies available for carrying out data science projects. For example, CRISP-DM, SEMMA and KDD are general (industry-developed) process models. [2,13,14]. Such processes include relevant steps that are considered to be standard for analytics, data mining and data science projects. The Data Science Trajectories (DST) framework [3] is related to these earlier models and has the unique possibility of detailed adaption of its approach. Adapting the approach composes a so-called trajectory and might enable to (better) suit specific types of projects.

This article outlines the process of applied data science research across multiple underlying studies and its relation to traditional methodologies. We propose a generalised protocol based on the DST framework, detailing each step with key considerations. Using examples from a PA intervention study, we illustrate its practical application. Additionally, we review CRISP-DM and DST methodologies to assess the relative strengths of our proposed trajectory.

## Method details

### Data science methodologies

#### The classic CRISP-DM approach

**Fig. 1:**
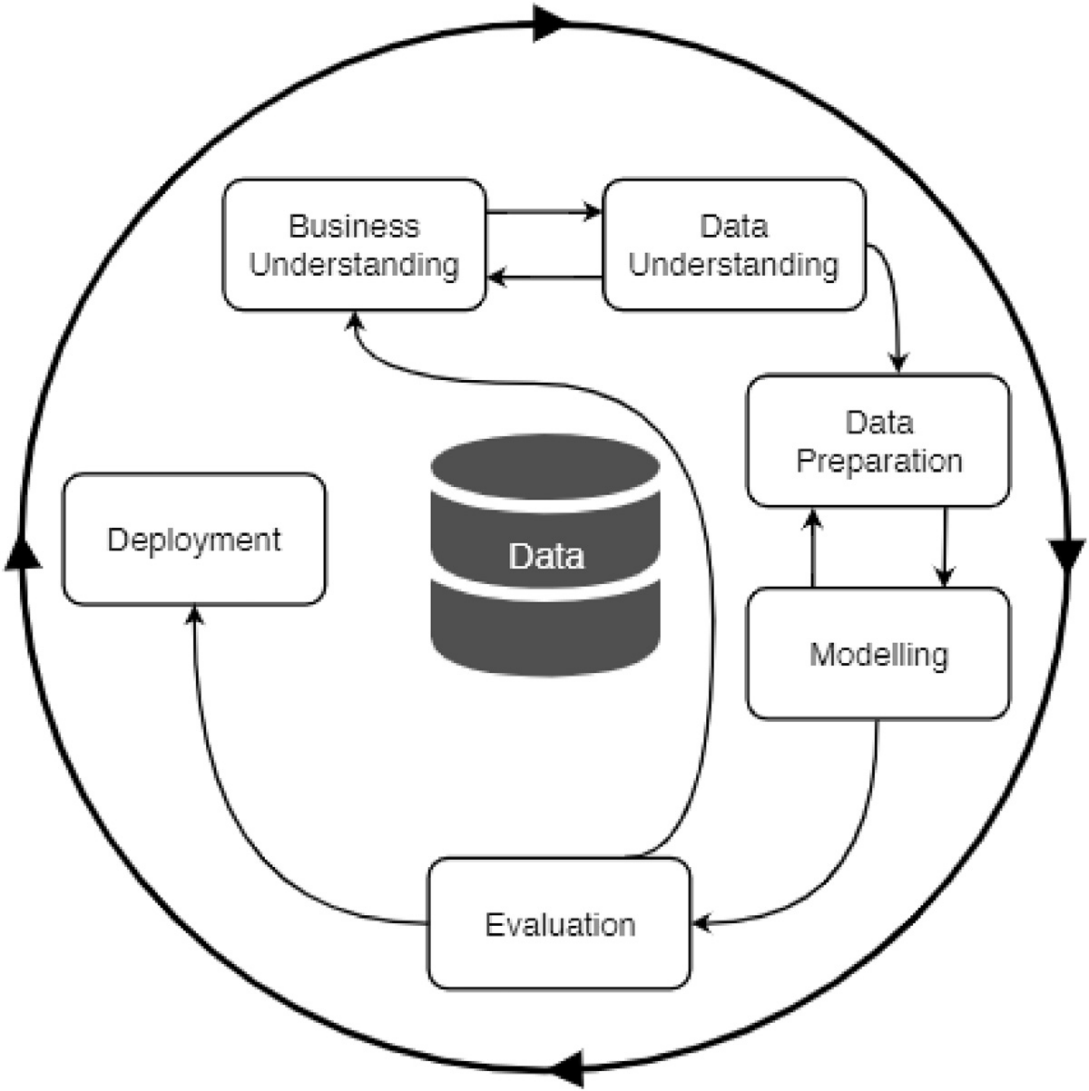
The CRISP-DM model [2].

CRISP-DM is a data mining methodology developed from a goal-oriented perspective, which originates from 1999 and from which several methodologies and models evolved, such as SEMMA [2,3]. In the view of CRISP-DM, data is a given means to achieve a certain goal and is already collected and available for processing. It focuses on the process of data analysis projects and includes six phases consisting of several generic tasks, which are depicted in Fig. 1. The sequence of phases is not rigid. Which phase to be performed next is dependent on the outcome of the current phase. The arrows in Fig. 1 indicate the most important and frequent dependencies between phases. The outer circle symbolizes the cyclical nature of data mining, meaning that lessons learned during a process can trigger new questions. The following are brief definitions of the phases [2], which are composed of different activities.

• Business understanding: focuses on understanding the project objectives and requirements from a business perspective.
• Data understanding: starts with an initial data collection and proceeds with activities to get familiar with the data.
• Data preparation: covers all activities to construct the final dataset, including tasks such as table, record and attribute selection as well as cleaning and transformation of the data, which are likely to be performed multiple times.
• Modeling: various modeling techniques are selected and applied, often with requirements regarding the form of data, and parameters are calibrated to optimal values.
• Evaluation: evaluate the model built (having high quality from data analysis perspective) and review the steps executed to construct the model to be certain it properly achieves the business objectives.
• Deployment: make use of the created models.

#### Limitations of CRISP-DM and alternative methodologies

The most commonly-known approach of CRISP-DM provides a quite linear or sequential approach and might not be flexible enough to accommodate projects that are less straightforward and more iterative, for example in applied data science research [15]. One can divert to another methodology that differs from this classical approach in certain respects. Within SEMMA more emphasis is put on data exploration, making it better suited to situations where exact analysis objectives have not yet been defined [16]. Within KDD, including the steps Selection, Preprocessing, Transformation, Data Mining and Interpretation/Evaluation, there is more emphasis on extracting useful, new knowledge from the data, rather than merely focusing on constructing a model that represents the data [16]. The DST framework is an iterative and exploratory approach, and differs from earlier approaches because it allows for customisation across the whole project duration [3].

#### DST

**Fig. 2:**
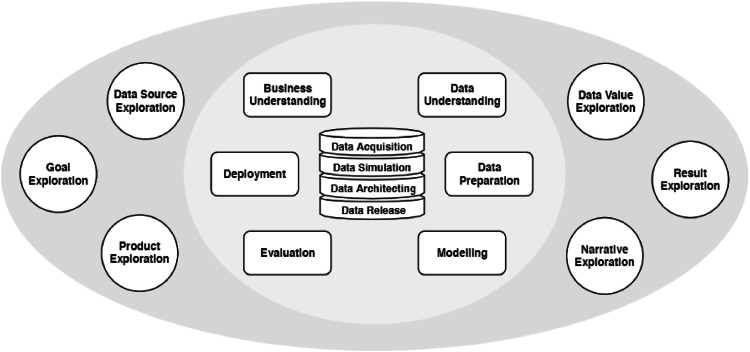
The DST map [3].

The Data Science Trajectories (DST) framework is derived from the original CRISP-DM methodology by extension with new activities in data science [3]. The authors outline the classic CRISP-DM as being too restrictive for the current data-oriented and exploratory perspective of (applied) data science, where value is extracted from data using scientific methods. The DST methodology is less prescriptive, including activities you can *possibly* perform on the data in an order that depends on the domain and decisions and discoveries by the data scientist. A data science project follows a particular so-called trajectory through a more broadly defined space including possible exploratory activities, CRISP-DM goal-directed activities and data management activities (Fig. 2). Note that the CRISP-DM phases are covered in the DST space, but are not necessarily executed (in the standard, pre-defined order). DST is broadly applicable; different trajectory charts can be distinguished, showing transitions of activities from the DST map for particular kinds of application contexts [3].

#### DST in the context of applied data science on multiple studies

We aim to provide an analysis procedure, describing the process and general steps to follow in applied data science research on multiple studies. It is appropriate to model this procedure within the DST framework. This not only allows adaptation to an exploratory approach towards the specification of detailed analysis objectives, but also refinement of resulting models to suit answering the research questions. Indeed, in the case of working with multiple studies, it is plausible that exact goals and hypotheses are not predefined, but are further specified as one obtains more insight into the way studies and associated datasets are related. Furthermore, collaborating with multiple disciplines can lead to a situation where exact goals need to be adjusted in the first phase of the study, such as decisions about the inclusion of studies. Taking into account the original research questions, models might need to be tuned to match requirements from the application area.

### Physical activity behaviour change application

#### Case study details

The case that is taken in our paper as an example, in order to clarify how steps in the proposed methodology might look like, concerns a study into the behavioural change pathways through which the effects of PA interventions are achieved. To gain health-related benefits, several behavioural change interventions have been designed to stimulate PA by influencing relevant factors determining this behaviour (so called determinants of behaviour) [[17], [18], [19], [20], [21]]. The intervention content individual participants receive is tailored based on individual participant characteristics. Insight into the exact working mechanisms of these interventions could be used to enhance their effectiveness.

Single study analyses applying traditional analysis techniques have shown the effectiveness of interventions, but have not revealed insight on working mechanisms [7,8,[22], [23], [24], [25], [26], [27], [28], [29]]. The PA case study aimed to gain insight into the (sub-population specific) mechanism of PA behaviour change induced by (online) interventions, by data-based estimation of Bayesian networks [22] using the combined data from five different Randomized Controlled Trial (RCT) intervention studies [1].

#### Data description

The study data that have been enclosed in our case study are from the different studies, each focussing on a different target group [[17], [18], [19], [20], [21]]. In all studies, data have been collected for intervention participants and participants in a control group. The raw data includes variously operationalized determinants, demographic factors and PA outcomes (e.g. minutes of moderate to intensive PA behaviour per day). Data have been measured at different points in time, namely at the baseline and at 3, 6 and 12 months after the baseline, measuring intervention effects. However, some variables have not been measured at some time points in particular studies, creating (additional) missing values when integrating the data from these multiple studies.

### The proposed methodology for step-by-step analysis of multiple studies

This section starts with our proposed more general trajectory for applied data science projects in which multiple studies and datasets are included (to eventually analyse sub-populations). Subsequently, a detailed description of its steps and their execution is given, including core decisions and examples from the case study.

**Fig. 3:**
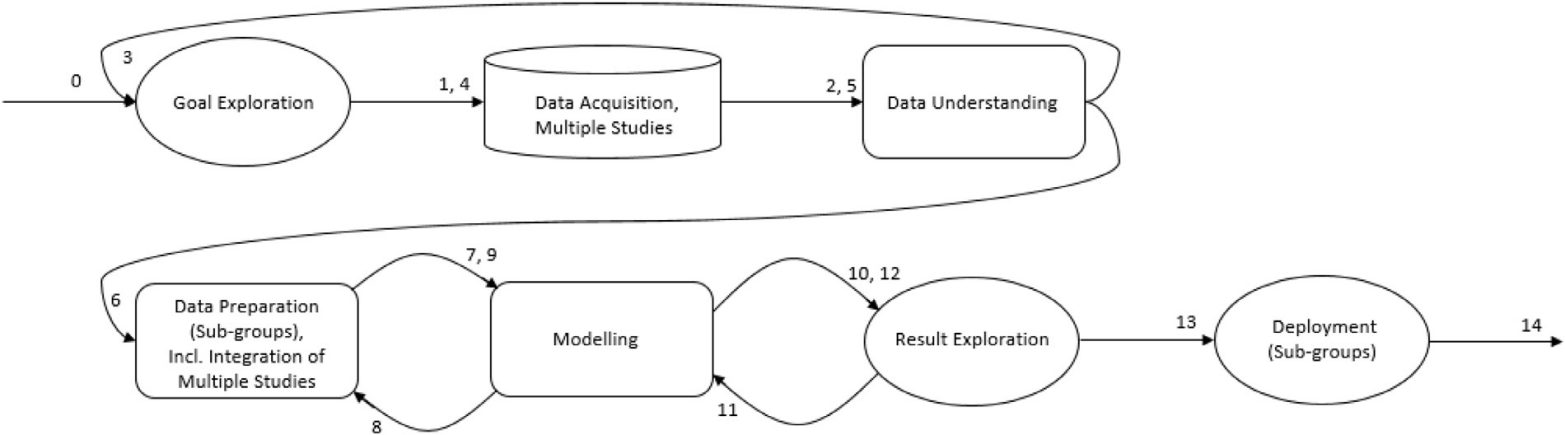
Proposed DST trajectory for data science analysis of multiple studies.

The sequence of activities we propose for projects within applied data science research with multiple datasets, such as our research, can be presented as a trajectory chart according to the DST framework [3]. This trajectory, as shown in Fig. 3, consists of 7 different activities and is dynamic in the sense that several activities are visited more than once. The numbers in the figure represent the order of transition between activities. Notice the reverted arrows, returning to a previous activity. This is dynamic, which means that whether and how often to go back depends on the execution of the current activity. As in every project, the activities are accompanied by obstacles and decision points. In this section, we further explore the implementation of activities, and outline key considerations and the way they have been addressed for the particular context of the case study. An overview of the key considerations discussed for each step of the proposed protocol can be found in Table 1.

**Table 1:** Overview of key considerations for protocol steps.

Protocol step	Key topics
Goal exploration	- Establish understanding of background - Exploration of field-specific terminology and jargon
Data acquisition	- Collection of data - Selection of relevant datasets for analysis
Data understanding	- Establish understanding of data context and assumptions
Data preparation	- Documentation of metadata - Data cleaning - Handling missing values - Standardisation of variables across different studies
Modelling	- Selection of suitable analysis technique - Learning model structure and hyper-parameter optimisation - Learning model parameters
Result exploration	- Assess model reliability and stability - Visualisation of results to address research questions - Identification of patterns and interpretation of key relations
Deployment	- Providing customised recommendations

#### Goal exploration, data acquisition and data understanding

The first 3 activities are closely related to each other and are therefore discussed together. A project starts with clarifying the theoretical background. Also, the data scientist needs to become familiar with (technical) jargon of the specific application area, which is crucial in many multidisciplinary data science projects. In our research, the field of application was health psychology. To understand the data, besides literature research, conversations were held with researchers of studies to be (possibly) included. During such conversations, the context and assumptions of the data can be discussed in order to decide which data to be analysed, possibly focussing on specific vulnerable groups. For example, based on the characteristics of various studies, a selection of datasets is made which are amendable to data fusion. Related to jargon, we encountered the terminology of item and concept, to indicate specific variables, which typically have a different meaning in data science.

Once the objectives or research questions are clear, relevant data can be obtained from multiple included studies, in the *data acquisition phase*.

After acquiring the data, the data scientist ought to examine data properties to create an overview of content-related and technical details. Fundamental when working with domain experts, is clarifying the semantic of variables in order to avoid inconsistency. From a big data perspective, in case of dealing with multiple studies, it is important to know which data has been measured in which study at which time point. In order to be able to make decisions about transformation and integration of data for cross-study analyses, it is important to know how variables are measured in each included study. For example, in the case study, the questionnaire items from five included studies that were to be translated into a concept, their answering options and corresponding values, were important. From a technical point of view, in the data understanding phase, user- and system-missing values and the magnitude of the missing value problem are relevant to explore in depth.

Once there is a good understanding of the available data and gaps in existing literature, decisions can be made about the integration of data to be analysed across studies, the concretisation of research question(s) to be answered and how to address them data-driven. Examples of decisions regarding data integration are the transformation of variables to overarching definitions across studies, how to deal with the fact that variables might not be measured in all included studies and at similar time points, and inserting an index variable to indicate in which study the record was measured. The sharpening of research objectives (*goal exploration*) can lead to adjustments in *data acquisition* (and *data understanding*), for example by requesting additional data or data in specific formats to facilitate the integration process.

#### Data preparation

Proper data preparation and documentation of meta-data form the basis for the modeling phase that follows. The data preparation phase mainly consists of cleaning and transforming the data, just as in the CRISP-DM model [2]. When dealing with multiple studies, carrying out the data integration is a central aspect of this phase.

The data preparation phase also takes into account future research. To enable the re-use of data for future research as much as possible, the set of principles Findable, Accessible, Interoperable and Reusable has been drawn up that should also be followed [30].

The modeling phase of a data science project is a dynamic process, which gradually expands and in which you might run into several obstacles, such as different types of missing data and reproducibility of resulting models. Some of the decision points encountered are common to data science projects, others are more specific to the application or the applied analysis technique in the specific project. In the remainder of this sub-section, we outline some key points and the way they have been approached in the case study.

As one of the first core considerations, one decides upon the most appropriate analysis technique to be applied and which software to be used to perform the analyses. For example, in our case study, we chose to analyse the data using Bayesian networks [22], because of its advanced, explorative approach to discovering the probabilistic dependence structure among variables, which can be represented graphically as directed acyclic graph [22]. More formally, given a graph *G* learned from the data, two variables are directly influencing each other if there is an arc between the variables in *G*. Similarly, *Z* mediates the relation between variables *X* and *Y* if there is a path from *X* to *Y* through *Z* in *G*. This method helps research in representing and reasoning with uncertain knowledge. In the case study, it was decided to perform these analyses in R, as it lends itself well to performing advanced analyses. Moreover, one can make use of an existing well-documented and comprehensive package to learn and perform inference on these models [31].

As shown in the trajectory in Fig. 3, there is a step from the modeling phase back to the data preparation phase. This can be because of requirements of analysis techniques on the form of the data, or the determination of a relevant sub-selection of the prepared data to be explored and associated cleaning operations. For example, in the case study, the selection was based on theoretical arguments about the PA behaviour change mechanisms to be modeled. Also technical reasons played a role, such as requirements regarding data availability due to missing data and the number of studies in which certain variables are measured.

In scientific research, different facets of a phenomenon might be examined step-by-step, where for example sub-populations are investigated in more detail. Moreover, merging data may give different insights than from the corresponding separate subsets [32]. Note that in research across multiple studies sometimes not all measurements are assessed in the different sub-studies. This leads to empty variables, where all records of a specific variable are missing. During data wrangling, a subset of prepared samples is sometimes chosen. This can result in the data of a variable being entirely missing or constant, and is more likely to occur in sub-population research. For example, in the PA case study, the effect of determinants was investigated in sub-groups. In some sub-groups some variables have not been measured or are deterministic. For applying machine learning, the variable has to be removed from the dataset for that sub-population.

#### Modeling

As soon as no more adjustments need to be made in the selection of prepared data to be included in analyses, the modeling phase can actually take place. Methodologies for data mining do not discuss the application of modeling techniques, and the challenge of optimal setting of their parameters and hyper-parameters in detail [2,3,16]. We distinguish a few key considerations to be relevant for most data science projects in general, namely: missing data handling, estimation of model structure and parameters, and reproducibility and reliability of analysis results. Therefore, we discuss these, including example approaches to them from the case study.

Dealing with missing data is an important topic in any real-world data science project. Several deletion and imputation methods are commonly used, based on different assumptions about the nature of the missing data such as being Missing At Random (MAR) [33]. It is important to first find out what is the nature and extent of the missing value problem within a specific project, and from there decide on the appropriate method. Moreover, missing values affect other considerations. For instance, parameters can be calculated for example from complete cases or the whole imputed dataset. Note that, when calculating certain test statistics from complete cases, it is important to consider sensitivity to the remaining sample size [34]. In the case study, one had to deal with a lot of missing data. Thereto, in an earlier research phase, the researchers compared the performance of different methods by means of cross-validation and based on these results, the decision was made to apply the Structural Expectation Maximisation (SEM) algorithm to handle missing data [[35], [36], [37]]. SEM, which is often used, iteratively combines the estimation of missing values with structure and parameter learning of Bayesian networks [36].

Learning a probabilistic model may involve both structure learning and parameter learning. In structure learning of a statistical model, the associations between variables are discovered. An important first step is to determine what kind of model is appropriate and the approach to learn its structure. In a knowledge-based approach, the structure is determined based on knowledge of experts in the field, whereas in the data-driven approach this is based on information following from a given dataset [38]. There is also a hybrid approach between these two extremes, sometimes called knowledge-based learning, combining information from experts with information from the data. In the case study, (temporal, hybrid) Bayesian network models were decided to be learnt data-driven in order to obtain insight into dependency structures of a selection of important variables [[39], [40], [41]]. In order to do so, there are different classes of algorithms available [42]. A search-and-score-based algorithm (tabu search) was chosen, and, because of that, the decision had to be made of which model score to be optimised (BIC) [[42], [43], [44]].

During parameter learning the given data are fitted to the model to estimate configuration variables that are internal to the model [45]. Important assumptions here are the type and distributions of variables in the model. For example, whether it is a discrete, continuous or mixed model. Under these assumptions, one can decide to calculate for example maximum likelihood estimates or mutual information estimates [[46], [47], [48]]. Several considerations may come across to play a role in this decision, such as missing data or interpretability. Because of the interpretation of parameters in a hybrid context, in the case study mutual information estimates were computed based on complete cases of included variables.

Reproducibility of results is an important standard in academics in general [49]. Obviously, it is recommended to properly document particular decisions made and coding scripts.

**Fig. 4:**
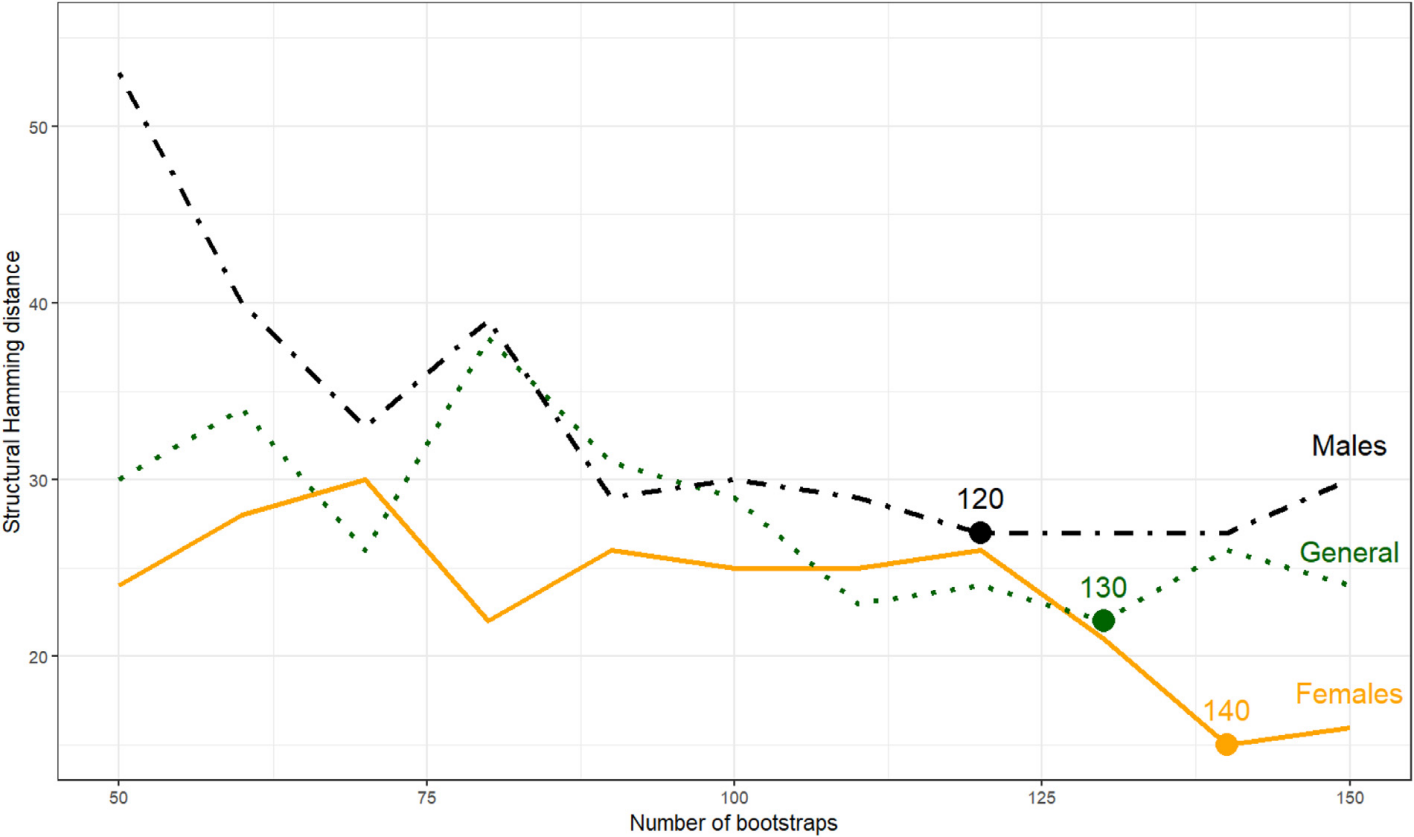
Stabilization of averaged Bayesian network models during bootstrap procedure in case study research [1].

Another important value in research is the reliability of the resulting models, i.e. the extent to which they are sensitive to data anomalies. This is specifically important if the number of possible models is large or there is a stochastic factor in the learning procedure such as with SEM. To this end, one often reports the stability of relations and models, which indicates the degree of certainty that specific relations occur and are learnt from the data. To investigate the stability of relations found, bootstrapping can be applied. Note that one should be aware of avoiding the creation of identical samples when resampling the dataset. In machine learning, one typically learns a so-called original model and compares this with the bootstrap outcomes [50]. An important decision hereby is the number of bootstrap samples reaching an intended level of stability. Another important decision is the data-driven threshold value, i.e. the minimum percentage of bootstrap sample models in which an arc has to occur to be included in the averaged model. In this stability issue, differences in approaches across disciplines occur. Namely, to obtain insights into the most important relations between variables, a data-driven threshold is fairly low. This does not necessarily match expectations in a scientific setting. In the case study, the models derived from repetitions of the experiment differed substantially with respect to goodness of fit (BIC),implying model instability [51]. Bootstrapping was applied to learn the model structures. Due to the extent of difference between models learned from for different resampled datasets, the researchers decided to deviate from the standard approach in machine learning and defined the averaged model based on the percentage of bootstrap sample models in which certain relations occur in a certain direction. Assuming that there is always some instability left over due to the large amount of imputed missing data, they stopped adding bootstrap samples as soon as the stability of the averaged model no longer significantly improves (based on the structural Hamming distance) [52,53], see Fig. 4. A summary of the approach on modeling considerations in the case study is provided in Table 2.

**Table 2:** Summary of modelling parameter details in the case study.

Aspect	Considerations	Example approach from case study
Missing data	Assess the nature (e.g. MAR) and extent; select appropriate deletion or imputation method	Cross-validation of methods for optimal handling of extensive missing data; applied SEM
Model structure	Chose knowledge-based, data-driven or hybrid approach; decide on technique, learning algorithm and scoring function	Data driven; (temporal, hybrid) Bayesian network models trained using tabu search with BIC as scoring function
Parameter estimation	Assess variable types and distributions (discrete, continuous or mixed); choose estimation method	Hybrid context; mutual information estimates from complete cases for reasons of interpretability
Reliability	Check sensitivity to data anomalies; ensure model stability; determine threshold for arc inclusion	Bootstrapping to assess stability; ensured stability of averaged model by adding samples until reached threshold (structural Hamming distance); stability of arcs as model parameters

#### Result exploration

Result exploration is meant to disclose the information necessary to answer the research questions [3]. In data science it is common to search for a resulting model suitable to answer the key research question(s), where interpretability and visualisation play a role [54]. Note that this differs from the way of approaching outcomes in applied fields, such as psychology. Here, one takes resulting models as premise and test for confirmation of the hypotheses. The search for the outcome desired to be able to properly answer specific research questions defined earlier sometimes causes important changes for the modeling phase. Therefore, the proposed trajectory represents a link from result exploration back to the modeling phase. In the case study, the step of result exploration mainly consisted of distilling paths relevant with respect to answering the research question, from large (averaged) Bayesian network models (derived from the bootstrap procedure), and customizing their visualization. There were also some links back to modeling in the case study. For example, in choosing the stability threshold and in the consideration of several ways to distil relevant paths, the interpretability of the resulting Bayesian network model fragments was considered. This meant that, by distilling the most stable and relevant paths, the researchers aimed to find sub-models based on which they could find answers to main questions and that were simplified enough to draw conclusions from them. Further, for reasons of interpretability, it was decided to quantify the strength of relations by jack-knife bias-corrected mutual information estimates [47]. Since visualisation can contribute to the interpretability of results, nodes have been coloured and model parameters got interpretable representations, for example by asterisks. From the representations of stable, relevant Bayesian network model fragments, new knowledge was revealed about roles of determinants in affecting short- and long-term PA. The network model presents the complex interplay between determinants in influencing and maintaining this behavior. Further, in subpopulation-specific analyses, which particularly benefit from integration of data from multiple studies, differences are shown in relevance and roles of determinants.

#### Deployment

Once suitable results are obtained, the final step is to interpret them in context of the application domain. In applied data science, one tries to answer content-related research questions. Here it can be beneficial to reach conclusions in consultation with the field. In sub-population analyses, interpretation can for example imply comparison of models estimated based on relevant sub-sets of the data. In the case study, based on the resulting models, advice was issued on how to adapt interventions for specific vulnerable sub-populations to increase their effects.

### Method validation: a comparison of the proposed trajectory and standard CRISP-DM methodology

To evaluate the relevance of our trajectory for analysing multiple studies, we elaborate on differences as well as similarities between the classic CRISP-DM methodology and the proposed adapted process. The classic CRISP-DM methodology is intended to be applied to solve business problems using data science [2], and is therefore likely to show differences from our proposed trajectory. The approaches to specifying objectives and dealing with results in our protocol are described to be more explorative in our trajectory and similarities are found in other important core activities, e.g. in data preparation and modelling.

### Differences

The most important differences between the proposed procedure and following standard methodologies such as CRISP-DM occur in the first phases of research and in the way resulting models are approached.

While there are global objectives and research questions defined, the exact objectives and requirements are not pre-defined in the proposed methodology, whereas in CRISP-DM one starts from a more pre-defined business goal. Data from multiple studies means dealing with different contexts and assumptions, e.g. different target populations. This, and the multidisciplinary nature of data science and its application domain complicates the path to accomplishing precise research goals. In order to decide about study-overarching analyses, differences and similarities between contexts of multiple datasets need to be fully clear. Through preliminary research into the theoretical backgrounds of the data and consultations with experts in the domain, the data scientist can get a clear sense of the phenomenon that is intended to be modelled and existing knowledge. Differences in study design and the measurement data are especially important here (e.g. in our research the differences in operationalisation of psychosocial constructs through which effects are hypothesized to occur). After creating a comprehensive overview of the differences and similarities between the datasets, decisions can be made about how the data can be analysed at a higher level, across studies, and which studies and data to be included in an integrated study (possibly to analyse specific sub-populations). Instead of starting from project objectives from a business perspective and getting familiar with initial data, as in classic methodologies, in the proposed methodology detailing of objectives is being performed explorative. This results in these interactions with phases of data acquisition and data understanding. Following the traditional CRISP-DM methodology, one needs to check whether the resulting model is suitable to properly reach the defined objectives [2]. Related to that, there is a difference in way of thinking from an application domain point of view, as experienced in the case study application in the psychology field, and from a data science point of view [55]. Within empirical research such as is common in psychology, one works hypothesis-driven and test for confirmations in the data. Within machine learning, one works data-driven in search of an answer to a research question, or an explanation for a phenomenon or a pattern. From that, in our proposed methodology, models and visualisations can be refined (iteratively) in search of a suitable result (result exploration). A summary of the discussed differences between the proposed and classic CRISP-DM methodologies is provided in table 3.

**Table 3:** Summary of differences between the proposed trajectory and CRISP-DM methodology.

Aspect	Proposed trajectory	CRISP-DM methodology
Objective definition	Broad research question, detailed objectives evolve through exploration and preliminary research	Predefined business goal
Context of data	Focus on integrating data from multiple studies, with diverse contexts, assumptions, and target populations	Primarily single-context data
Data preparation	Includes integration of datasets and transformation to align with study-overarching variable definitions	
Model evaluation	Iterative refinement of models and visualisations	Models evaluated against predefined objectives

### Similarities

The data preparation and modelling steps align with the standard CRISP-DM methodology. However, in the proposed methodology, the transformation of data has a more specific focus on the integration of datasets and the determination of a relevant sub-selection of the data (for example, to explore sub-groups). In particular, the integration takes place according to rules defined in previous steps of data understanding and goal exploration. For example, in the case study, relevant concept variables were derived from the raw item data according to the study's overarching concept definitions. Even when largely following the standard methodology, also the modelling phase is typically accompanied by specific decision points, often related to the chosen analysis method. Note that the analysis technique also affects the execution of the preparation phase, by putting requirements on the form of the data.

Apart from decisions that are specific for a particular study, in the context of multiple studies and multidisciplinary data research the determination of research focus and approach of searching for associated appropriate results is complex. Following the differences and similarities, the conclusion can be drawn that a more specific methodology enables this type of data science research more than a standard methodology.

## Conclusions

Data fusion is known to have advantages with regard to supporting data-driven analyses. This is especially important for analyses within sub-groups or in data from studies that have small sample sizes. By analysing data from multiple studies in an integrated way using appropriate data science techniques, one can gain more insight into relevant mechanisms. For example, insights into mechanisms leading to a healthy lifestyle are needed to develop eHealth tools to support this behaviour. This paper provides a specific methodology for such complex multidisciplinary data science study of multiple studies, which can be applicable to various application areas. Existing methodologies for data research are often developed from the perspective of industry and are quite general [2,3,16]. To the best of our knowledge, this is the first study that proposes such a methodology in a specific, complex (scientific) context.

In addition, we have discussed the steps of this methodology in detail. We elaborate on important decisions involved and provide examples of their concrete implementation in a PA intervention study applying the Bayesian network model. There are studies in the literature that highlight certain considerations in data research, such as methods for dealing with missing data, or different algorithms for training models such as for learning Bayesian networks [33,42,43,45]. However, there is a lack of studies that elucidate both these kinds of major considerations and other, often smaller ones, as part of steps in an analysis methodology. Since existing methodologies describe associated steps more globally, the comprehensive descriptions we propose can be a relevant guide for future projects.

The added value of examining an integrated dataset has been demonstrated in previous studies, such as that of the multidisciplinary PA case study [1,35], and multidisciplinary research is expected to continue to rise in the coming years. We aligned our proposed methodology for this kind of research with a standard, commonly known methodology on data research (developed for and from an industry perspective) and discussed differences and similarities. The comparison of these procedures revealed that standard data science approaches such as CRISP-DM are not necessarily suitable for this kind of research. Therefore, the presented methodology is likely to add value in a wide range of domains.

The detailed described protocol is expected to be particularly useful for researchers within (the intersection of) the data science field and applied domains. Note that application of the proposed methodology is not straightforward. Intensive involvement from the data science, as well as the application area, remains important in carrying out the steps, especially for recognising and solving (technical) issues and considerations.

Regarding the concrete implementation, there are contextual possibilities to extend detailed step descriptions in future. One can imagine that for some multidisciplinary compositions, issues may be experienced that have not yet been considered. In addition, for some types of analyses, there could be other types of considerations not discussed in this paper. To guide to a wide range of (scientific) audiences, the methodology could be evaluated continuously within other applied disciplines and descriptions extended accordingly.

To conclude, our currently proposed methodology provides guidelines to perform analyses with multiple datasets, which is particularly valuable for investigating complex phenomena in specific sub-groups. We expect that the guidelines and considerations discussed in this paper can guide future data science research in various application domains.

## Limitations

Not applicable.

## Ethics statements

The work did not involve human beings, animals or data collected from social media platforms.

## CRediT authorship contribution statement

**Simone Catharina Maria Wilhelmina Tummers:** Conceptualization, Investigation, Methodology, Project administration, Writing – original draft, Writing – review & editing. **Arjen Hommersom:** Conceptualization, Funding acquisition, Methodology, Supervision, Writing – review & editing. **Catherine Bolman:** Funding acquisition, Supervision, Writing – review & editing. **Lilian Lechner:** Funding acquisition, Project administration, Supervision, Writing – review & editing. **Roger Bemelmans:** Funding acquisition, Writing – review & editing.

## Declaration of competing interest

The authors declare that they have no known competing financial interests or personal relationships that could have appeared to influence the work reported in this paper.

## Data Availability

No data was used for the research described in the article.
